# Neuroimaging during Trance State: A Contribution to the Study of Dissociation

**DOI:** 10.1371/journal.pone.0049360

**Published:** 2012-11-16

**Authors:** Julio Fernando Peres, Alexander Moreira-Almeida, Leonardo Caixeta, Frederico Leao, Andrew Newberg

**Affiliations:** 1 Division of Nuclear Medicine, Department of Radiology, University of Pennsylvania, Philadelphia, Pennsylvania, United States of America; 2 Center for Spirituality and the Mind, University of Pennsylvania, Philadelphia, Pennsylvania, United States of America; 3 PROSER – Institute of Psychiatry, Universidade de Sao Paulo, Sao Paulo, Sao Paulo, Brazil; 4 Research Center in Spirituality and Health, School of Medicine, Universidade Federal de Juiz de Fora, Juiz de Fora, Minas Gerais, Brazil; 5 School of Medicine, Universidade Federal de Goias, Goiania, Goias, Brazil; 6 Myrna Brind Center for Integrative Medicine, Thomas Jefferson University, Philadelphia, Pennsylvania, United States of America; University of Cardiff, United Kingdom

## Abstract

Despite increasing interest in pathological and non-pathological dissociation, few researchers have focused on the spiritual experiences involving dissociative states such as mediumship, in which an individual (the medium) claims to be in communication with, or under the control of, the mind of a deceased person. Our preliminary study investigated psychography – in which allegedly “the spirit writes through the medium's hand” – for potential associations with specific alterations in cerebral activity. We examined ten healthy psychographers – five less expert mediums and five with substantial experience, ranging from 15 to 47 years of automatic writing and 2 to 18 psychographies per month – using single photon emission computed tomography to scan activity as subjects were writing, in both dissociative trance and non-trance states. The complexity of the original written content they produced was analyzed for each individual and for the sample as a whole. The experienced psychographers showed lower levels of activity in the left culmen, left hippocampus, left inferior occipital gyrus, left anterior cingulate, right superior temporal gyrus and right precentral gyrus during psychography compared to their normal (non-trance) writing. The average complexity scores for psychographed content were higher than those for control writing, for both the whole sample and for experienced mediums. The fact that subjects produced complex content in a trance dissociative state suggests they were not merely relaxed, and relaxation seems an unlikely explanation for the underactivation of brain areas specifically related to the cognitive processing being carried out. This finding deserves further investigation both in terms of replication and explanatory hypotheses.

## Introduction

Dissociation is typically defined as the lack of normal integration of thoughts, feelings, and experiences into consciousness and memory [1]. The idea that traumatic experiences cause dissociative symptoms is a recurrent theme in clinical and neuroimaging literature, and some of the cognitive phenomena associated with dissociation appear to be dependent on the emotional or attentional context [2], [3]. Although non-pathological dissociation is quite common in the general population, dissociative experiences are mostly studied as a risk factor for dissociative pathology [4], [5]. Spirituality and religiousness have been shown to be highly prevalent in patients with schizophrenia and dissociative symptoms [6]. However, the varying methodological issues and discrepancies among the studies developed so far make it difficult to articulate a comprehensive framework for brain activity and cognitive mechanisms in pathological and non-pathological dissociation.

Although the nature of the mind and its relationship with the brain is still one of the most challenging issues for science [7]–[10], assumptions made in this respect are the cornerstones guiding therapeutic interventions [11]–[13]. This study addresses important theories underpinning creativity and include religious and spiritual experiences. The American Psychiatric Association [14] pointed to the need for more research in this field by recognizing the non-diagnostic (non-pathological) category of “Spiritual and Religious Problems” in the DSM-IV, thus healthy forms of dissociation [15], [16] may be distinguished from pathological ones [2], [5].

Mediumship, a spiritual phenomenon that has often been reported throughout human history, is defined as an experience in which an individual (the medium) claims to be in communication with, or under the control of, the mind of a deceased person or other nonmaterial being [17]. Mediumistic experiences are usually dissociative, such as motor, sensory or cognitive automatisms (e.g. hearing spirits or reporting body movements or thoughts caused by spirits), and alternate identity or possession). Therefore it is no surprise that the study of mediumistic experiences was crucial to the development of ideas concerning unconscious and dissociative processes. Pierre Janet's classic 1889 study of dissociation examined several mediums; Carl Jung's doctoral thesis was a case study, and William James did meticulous research on the medium Leonore Piper [18], [19]. There has been a trend to divide dissociation in two broad categories: detachment (a sense of separation from the self or the world) and compartmentalization (inability to deliberately control actions or cognitive processes that would normally be amenable to such control) [20]. Although it sometimes involves detachment too, mediumship usually relates to the compartmentalization subtype.

Psychography is one of the many possible dissociative forms of mediumistic expression [17]. “Writing mediums” or psychographers claim that they write under the influence of spirits, and some pyschographed writings have had a major impact in different communities around the world. Brazil's most significant and prolific psychographic medium, Chico Xavier, whose education ended with elementary school, produced over 400 books of automatic writing spanning a wide range of styles and subjects, selling several million copies, with all copyright earnings donated to charity [21], [22].

A study of the mental health of 115 spirit mediums [17], [23] found that subjects had high socio-educational levels, showed low prevalence of psychiatric disorders, and were well adjusted socially compared with the general population. Their experience of mediumship was distinct from dissociative identity disorder. Nevertheless, few studies have investigated the neural substrates underlying dissociative states of consciousness related to religious experiences [24]–[26]. In one previous neuroimaging study of glossolalia – a trance-like state with vocalizations that sound like language but lack clear linguistic structure – subjects were found to have reduced activity in the left caudate nucleus and the right prefrontal cortex, along with increased activity in the superior parietal lobes [25]. Neurofunctional research on sensitive experiences such as religious ones requires specific methods that do not adversely affect volunteers' performance [27].

Like the glossolalia study, the present study utilized single photon emission computed tomography (SPECT) to measure regional cerebral blood flow (rCBF), which is closely correlated with brain activity. We used the SPECT neuroimaging method for this study because it enables researchers to maintain a suitable environment free of distracting/ansiogenic effects for subjects performing complex tasks requiring silence and concentration. To our knowledge, there have been no previous studies of the association between claimed mediumistic dissociative states and specific CBF alterations.

Based on our prior research on related practices such as meditation and prayer, we focused primarily on the prefrontal cortex and anterior cingulate gyrus since both are known to be involved in the brain's attentional network [24], [25]. Furthermore, these areas are involved, along with Broca's area, in the production of speech. We also found evidence of changes in thalamic activity in limbic structures such as the hippocampus, and the superior temporal region is involved in a number of processes including language reception. The precentral gyrus may be involved in the motor function related to writing. Therefore, our hypothesis-driven analysis focused on these regions.

We studied the neurophysiological nature of dissociative mediumship in psychography as measured by changes in rCBF. During psychography, individuals write legible structured narratives but often claim to be unaware of the content or grammar of the written text. The present study aims to determine whether this type of dissociative trance state is associated with specific alterations in brain activity that differ from those found when writing normally, i.e. not in a dissociative trance state. Since psychographed contents feature complexity and planning, our *a priori* hypothesis was that the areas involved in cognitive processes while writing consciously, such as reasoning and planning content, would show similar activation during mediumistic trance.

## Methods

We examined 10 Brazilian psychographers who had been doing automatic writing for 15 to 47 years, producing 2 to 18 psychographies per month (Table 1), whom we divided into 5 ‘less expert mediums’ and 5 with ‘substantial experience. All were white, right-handed, in good mental health (Table 2), and not currently using psychiatric drugs. The criteria used to describe mediums as ‘experienced’ was at having practiced mediumship for least 20 years and produced at least 10 psychographies per month at the time of beginning of the study.

**Table 1:** Sociodemographics.

Subject	Age	Marital status	Work status (not related to mediumship)	Educational level	Gender	Years of mediumship/psychographies per month
Exp 1	50	Married	Full time	University	Male	40/16
Exp 2	59	Single	Retired	University	Male	42/18
Exp 3	45	Single	Full time	University	Female	34/16
Exp 4	53	Married	Full time	University	Male	47/16
Exp 5	33	Married	Part time	University	Female	24/12
Lex 6	58	Divorced	Full time	University	Female	22/4
Lex 7	50	Single	Retired	University	Female	45/2
Lex 8	50	Married	Full time	High school	Male	25/8
Lex 9	40	Married	Housewife	High school	Female	15/2
Lex 10	45	Divorced	Full time	University	Female	5/8

Note: Experienced subjects (**Exp**) and Less expert subjects (**Lex**). **University**: bachelor degree.

**Table 2:** Mental Health data.

Subjects	SRQ	BDI	BAI	SAS-SR	Current mental disorder-SCAN	Borderline Personality disorder–DSM IV	Childhood abuse
Exp 1	4	6	6	1.67	0	0	0
Exp 2	0	0	1	1.58	0	0	0
Exp 3	1	0	0	1.65	0	0	0
Exp 4	2	7	7	1.82	0	0	0
Exp 5	1	5	2	1.69	0	0	0
Lex 6	2	3	4	1.57	0	0	0
Lex 7	2	3	2	1.39	0	0	0
Lex 8	5	6	14	1.91	0	0	0
Lex 9	0	0	0	1.37	0	0	0
Lex 10	4	1	1	1.41	0	Yes	Yes

Legend: Scores of experienced subjects (**Exp**), less expert subjects (**Lex**), on the Self-Report Psychiatric Screening Questionnaire (**SRQ**) (cutoff point for common mental disorders: >4 for men and >6 for women), Beck Depression Inventory (**BDI**) (cutoff for depression: ≥10), Beck Anxiety Inventory (**BAI**) (mild anxiety: ≥10–18, moderate: 19–29) and Social Adjustment Scale (**SAS-SR**) (range: 1 [best] to 5 [worst]).

The 10 mediums were well adjusted in terms of their family, social and professional lives, and regularly helped people who had lost loved ones (Table 1). None of them were paid for their mediumistic activity, which they see as part of their mission of helping people. All of them reported spiritual experiences in childhood or adolescence. Both groups had the same mean age: experienced (48+−9.8 years) and less expert (48.6+−6.7). ‘Experienced’ mediums had practiced mediumship for 37.4+−8.8 years with an average of 15.6+−2.2 experiences of psychography per month, against the ‘less expert’ records of 22.4+−14.8 years and 4.8+−3.0 times respectively.

The number of participants required to determine the statistical power of the study was based on previous glossolalia-related research [25]. Several mental health inventories and qualitative assessments of subjective experience were administered. Depressive symptoms were assessed using the Beck Depression Inventory (BDI) [28], anxiety symptoms using the Beck Anxiety Inventory (BAI) [29], past and current mental disorders using Schedules for Clinical Assessment in Neuropsychiatry (SCAN) [30]. Borderline personality disorder and history of childhood abuse were based on data from the Dissociative Disorders Interview Schedule (DDIS) [31], and psychiatric morbidities were screened using the Self-Report Psychiatric Screening Questionnaire (SRQ) [32] (Table 2).

The local Human Research Ethics Committee in Brazil and the Institutional Review Board at the University of Pennsylvania authorized the study, and all participants signed informed-consent forms.

### Neuroimaging Procedures

We measured rCBF using SPECT during psychography (writing while in a dissociative trance state) and compared the data with those collected during normally conscious or non-trance writing (the control task). Both writing tasks were carried out in a quiet and dimly lit environment. Volunteers were asked to do psychography in the same manner as in their regular activity as mediums. All followed the same procedure: they sat on the chair where they would perform their tasks, said a prayer, closed their eyes, and concentrated. Usually, they were in a state of trance within a few minutes, and took up a pencil and started to write. Mediums reported entering a state of trance very easily and quietly. For the non-trance writing, in the same place, they were asked to write normally on their thoughts and on a similar subject to the one they usually wrote on during psychography.

After the psychography task, all subjects were asked if they had achieved the mediumistic state (contact with a deceased person), and were also asked to rate their level of mediumistic experience from 1 ‘poorly achieved’ to 4 ‘successfully achieved’. The order of tasks was randomized among subjects to avoid the sequence effect and the monitored interval between tasks ensured distinction between trance and non-trance states for psychography and control writing respectively. The use of SPECT imaging for the purposes of this study allowed for the evaluation of the trance state itself. SPECT studies of rCBF are performed in such a manner that the scans reflect what is occurring at the time of injection of the radioactive tracer, which was during the control writing or psychography tasks rather than afterwards. This technique is also used clinically to evaluate seizures in patients during the seizure itself, when injection is performed [33]. Subjects are scanned afterwards, but tracer distribution is not reversible once injected and taken up in to the brain. This enables imaging of the trance state itself.

Subjects began writing in the room and wrote for 10 minutes, at which time they were injected through the IV canulas (inserted in their left arms) with 7 mCi of ^99m^Tc-ECD. After writing for another 15 minutes, a researcher signaled to stop writing and they were taken to the SPECT scanner for a 40-minute scan.

Images were acquired on a triple-headed scanner (Trionix Research Laboratory) using high-resolution fan-beam collimators. Projection images were obtained at three-degree angle intervals on a 128×128 matrix (pixel size 3.56 mm×3.56 mm) over 360°. SPECT images were reconstructed using filtered back projection, followed by a low-pass filter and 1^st^ order Chang attenuation correction.

After the first writing-task scan, subjects returned to the room to perform the second task (psychography or control). After being observed performing the second task for 10 minutes, they were injected in the same way with 25 mCi of ^99m^Tc-ECD, without disturbing them. Subjects then continued to perform the second task for 15 minutes, and the session was then ended. Each subject was scanned (second writing-task scan) for 40 minutes using the same imaging parameters as above. Mediums' phenomenological experience during psychography and control task were assessed using a semi-structured interview just after the image scan acquisitions.

### Image Analysis and Statistics

The raw rCBF data were converted to ANALYZE format and preprocessed using SPM5 (Wellcome Trust Center for Neuroimaging, London) implemented in Matlab 7.10. The rCBF images from both writing tasks were then realigned with each other to correct for small shifts between scans using a six-parameter rigid body transformation with 4th degree B-spline interpolation. Images were then spatially normalized to the T1 weighted template provided by the Montreal Neurological Institute (MNI) by means of a least-squares approach and 12-parameter spatial transformation followed by estimating nonlinear deformations as implemented in SPM5 and smoothed using a full width 8 mm Gaussian filter at half maximum.

Preprocessed rCBF images from each subject were entered into a first-level analysis comparing the two groups (experienced versus less expert) and two conditions (psychography versus control). The images from each subject were then entered into an exploratory second-level group analysis in which a 2×2 Repeated Measures ANOVA (SPM5) was performed to determine the main group effects (experienced versus less expert) and condition (psychography versus control). Global intensity differences were corrected by using proportional scaling. The resulting SPM{F} map testing interaction effect was thresholded at p<0.05 (Z>1.64) and a cluster extent of 100 contiguous voxels. Identified clusters were then divided into anatomical regions using the Talairach Daemon database [34]. Finally a linear correlation model was applied to compare changes in complexity of written content to changes in CBF in the regions with significant differences between psychography and control state.

### Analysis of complexity of written content

After writing for 25 minutes without a break, written content was assessed by a Brazilian Language and Literature PhD with extensive experience of scoring compositions submitted for university entrance examinations using Analytic Assessment [35], [36], which weighs several characteristics or components of effective writing to provide an in-depth rating of writing quality and skills. The writing evaluated involved approximately 350 words relating to the period in which the brain was impregnated with tracer. This analysis was masked (blinded) so that the analyst did not know which group each volunteer belonged to. The following criteria were used to analyze written content: (i) punctuation, (ii) selection of lexical items and spelling, (iii) verb and noun concordance, and pronoun collocation, (iv) development of subject matter, (v) sentence structure and articulation between parts, and (vi) consistency. Scores ranged from 1 to 4 for each criterion as follows: (1) poor, (2) fair, (3) good, and (4) very good (Table 3). Content scores for the two groups were compared using the Wilcoxon Signed-Rank Test.

**Table 3:** Text Complexity.

	I	II	III	IV	V	VI	Total
**CT** Exp 1	3	2	3	2	2	2	14
**P** Exp 1	3	3	3	3	3	3	18
**CT** Exp 2	3	3	3	2	2	2	15
**P** Exp 2	3	4	4	3	3	3	16
**CT** Exp 3	2	2	2	2	2	2	12
**P** Exp 3	2	3	3	3	3	3	17
**CT** Exp 4	3	4	3	3	4	3	21
**P** Exp 4	4	4	4	4	3	4	22
**CT** Exp 5	3	3	2	2	3	2	15
**P** Exp 5	3	4	3	3	3	3	19
**CT** Lex 6	2	3	2	2	3	3	16
**P** Lex 6	3	3	2	2	3	3	21
**CT** Lex 7	2	3	2	2	2	3	14
**P** Lex 7	3	2	3	2	2	2	15
**CT** Lex 8	2	3	2	2	2	2	15
**P** Lex 8	3	3	2	2	2	3	15
**CT** Lex 9	2	2	2	2	2	2	12
**P** Lex 9	2	3	2	2	2	3	14
**CT** Lex 10	2	2	1	1	1	2	10
**P** Lex 10	1	2	2	2	2	2	11

Legend: The level of complexity of both types of written content (psychographed: **P** and control task: **CT**) was analyzed for each volunteer separately (Experienced: **Exp** and Less expert: **Lex**). Average complexity scores for psychographed content were higher than those for control-task writing, for both the whole sample [16.8 (SD 3.33) vs 14.4 (SD 2.95) - p = 0.007] and for experienced mediums [18.4 (SD 2.30) vs 15.4 (SD 3.36) - p = 0.041]. For less expert mediums, the difference was near significance [15.2 (SD 3.63) vs 13.4 (SD 2.41) - p = 0.066]. Planning for psychography writing was, on average, more sophisticated than for the control task, and the higher level of complexity relating to the more extensive planning work during psychography would require more activity from areas involved in cognitive processing.

## Results

Although the subjects studied had reported apparent delusions, auditory hallucinations, personality changes and other dissociative behaviors they did not present mental disorders and were able to use their mediumistic experiences to help others. Structured clinical interviews excluded current psychiatric illness. None of the subjects, except one with previous signs of borderline personality disorders, showed any clear sign of current Axis I or II mental disorders [14] (Table 2). All subjects stated that they felt very comfortable during the study and had successfully reached their usual trance state during the psychography task (4 ‘successfully achieved’), and this assessment was made shortly after the psychography task. All reported being in their regular/vigil state of consciousness during the control task. Seven found writing for the control task easy, and the three that mentioned some difficulty reported that they usually found it difficult to compose written texts in their everyday lives. During psychography, all mediums reported altered states of consciousness, but to different degrees. Experienced mediums spoke of a deeper trance, with clouded consciousness, often reporting being out of the body, and having little or no awareness of the content of what they were writing. Less expert mediums were in a less pronounced trance state and usually reported writing phrases being dictated to them in their minds.

**Figure 1:**
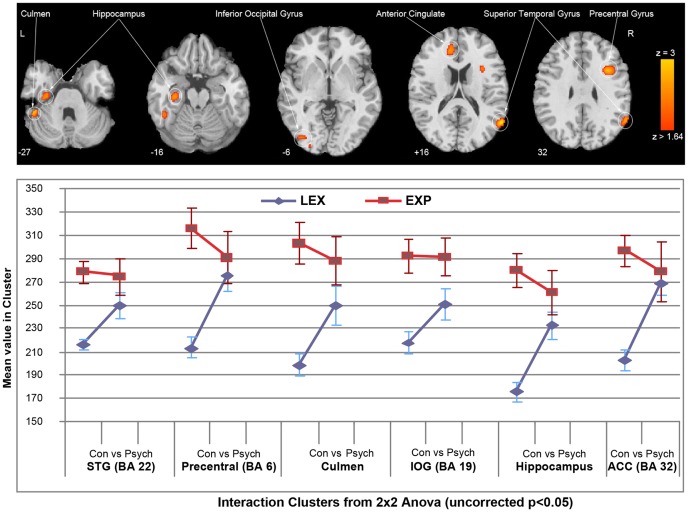
Results from 2×2 Repeated Measures Anova (SPM5) showing mean cluster size (sd) of the main effect of group (EXP: experienced - red vs LEX: less expert - blue) and condition (psychography vs control). The resulting SPM{F} map testing interaction effect was thresholded at p<0.05 (Z>1.64) and a cluster extent of 100 contiguous voxels.

Groups were randomized so there was no significant difference in mean time between scans. Using a t-test analysis of the regions based upon the counts per voxel, there were no significant differences when the entire group was analyzed. However, experienced subjects during the control condition showed significantly higher activity in these regions (p<0.001 for all regions) than less expert mediums. Significantly higher rCBF (p<0.01 for all regions) was shown in several areas of the brains of less expert psychographers, particularly in the left culmen, left hippocampus, left inferior occipital gyrus, left anterior cingulate, right superior temporal gyrus and right precentral gyrus (Figure 1, Table 4) during psychography compared to normal (non-trance) writing. The precentral gyrus focus actually spans the precentral gyrus and the medial frontal gyrus, but we reported the region based upon the MNI coordinates (Table 4). The experienced mediums writing in a trance state showed consistently lower rCBF in these regions than when writing in the control condition (Figure 1) – the difference was significant compared to the less expert ones (p<0.05). The written content produced by subjects during both types of task – with or without mediumistic trance – had never been written before. The level of complexity of both types of written content (psychographed and control-task) was individually analyzed for each subject. Content produced during mediumistic and control writing usually involved ethical principles, the importance of spirituality, and bringing together science and spirituality. The average complexity scores for psychographed content were higher than those for control writing (Table 3), for both the whole sample [16.8 (SD 3.33) vs 14.4 (SD 2.95) - p = 0.007] and for experienced mediums [18.4 (SD 2.30)3.36) - p = 0.041]. For less expert mediums the difference was near significance [15.2 (SD 3.63) vs 13.4 (SD 2.41) – p = 0.066].

**Table 4:** MNI coordinates for cluster centers of regions analyzed in Figure 1.

Region	Cluster CenterX Y Z
Superior Temporal Gyrus	58, −60, 18
Precentral gyrus	34, 12, 30
Culmen	−50, −42, −30
Inferior Occipital Gyrus	−40, −82, −2
Hippocampus	−34, −20, −20
Anterior Cingulate Cortex	−14, 38, 16

Finally, we performed linear correlation analyses comparing change in the overall complexity score for written content to change in CBF in the six regions identified as significantly associated with the psychography state. Overall, there was a trend towards an inverse correlation between change in complexity and change in CBF in each region. Correlation coefficients ranged from 0.59 to 0.74 for p values from 0.03 to 0.12. All correlations were inverse so that greater increases in complexity were associated with progressively decreased CBF in each region.

## Discussion

Our hypothesis was not confirmed for the less expert psychographers, as the results showed significant rCBF changes in several brain areas (Figure 1, Table 4) during psychography compared to non-trance writing. Moreover, contrary to our hypothesis, the experienced mediums doing dissociative writing in a trance state showed consistently lower rCBF in these regions than when writing in the control condition (Figure 1).

In relation to hypnotic suggestion, some studies showed prefrontal activation [37], but not others [38], whereas our subjects showed lower levels of activity in the frontal attention system. Although reduced frontal-parietal connectivity [39], and frontal deactivations are observed following a hypnotic induction in highly suggestible individuals [40], hypnosis is phenomenologically distinct from mediumistic expressions, therefore the two conditions are not directly comparable [41]. Moreover, the idea that hypnosis reflects a dissociative states remains controversial [42].

Brain scan studies of meditation have generally found increased frontal lobe activity and related attentional network [24], [43], [44], unlike our findings for the experienced mediums. Although meditative states do not necessarily involve dissociation and the phenomenological expressions are quite different from psychography, a recent study suggested that meditation improve the efficiency of brain functioning so that experts' brain activation levels are lower than those of less expert meditators [45], a pattern similar to that reported in the present study.

Previous neuroimaging research has shown that writing is a complex process requiring synchronized cognitive, language, and perceptual-motor skills [46]. Complexity of written content reflects the author's creativity and planning work underlying activity in the precentral gyrus, right superior temporal gyrus, left anterior cingulate, hippocampus, culmen, and occipital lobes. Damage or hypoperfusion in these regions has been correlated with severely impaired writing [46]–[48]. In particular, the experienced mediums showed higher complexity scores, suggesting that planning for psychographed content was more sophisticated than for content written while not in a dissociative mediumistic trance. The higher complexity of the text involving more creativity and planning work during psychography would presumably require more activity in the right precentral gyrus, right superior temporal gyrus, left anterior cingulate, left hippocampus, left culmen, and left inferior occipital gyrus [46]–[48] than would the less complex control task, but this was not the case, especially for the experienced mediums (Figure 1).

Findings concerning lower levels of left-hemisphere activity and higher right-hemisphere activity have been reported in pathological expressions of dissociative and psychotic experiences [49], [50]. Unlike our volunteers, schizophrenia patients had lower blood-flow levels in left-hemisphere regions, while higher-flow areas may reflect a need to draw on the right hemisphere to compensate for deficits in left-hemisphere networks [51]. Moreover, CBF abnormalities in anterior cingulate, precentral, temporal and culmen might be predictive for development of psychosis in high-risk subjects with subsequent transition to psychosis [50], [52], [53]. The anterior cingulate is involved in the attentional system in conjunction with emotional regulation, learning, memory, error detection, conflict monitoring, strategy planning, and empathy [54], [55]. Decreased anterior cingulate, precentral gyrus, superior temporal gyrus and hippocampus activity in experienced mediums may partly explain the absence of focus, self-awareness and consciousness during the dissociative state observed in pyschography. Despite several similarities with schizophrenic patients related brain activation [50], [52], [53], subjects participating in the present study did not have schizophrenia or any other mental disease (Table 2). This finding underlines the importance of further research into differential diagnosis between pathological and non-pathological dissociation [5], [16], [17].

We attempted to maintain as much similarity as possible between groups so that we could better compare their brain functions. The observed differences in CBF may be related to their different levels of expertise, but could also reflect differences in anxiety, effort or efficiency. For example, studies have shown that anxiety is associated with increased uptake in the ventral right prefrontal cortex and left insula/putamen area [56]. Thus, some of the changes we observed may have reflected anxiety, although none of the subjects reported particularly high levels of anxiety or stress.

Studies of cognitive expertise have revealed two general patterns of changes in cerebral activity. A number of studies have found that experts and non-experts show increased activity in different regions. The level of activity in the fusiform face area (FFA) when experts identify objects such as cars or birds, predicts performance on a behavioral measure of expertise made outside the scanner [57], [58]. However, studies have found that some brain regions show increases while others show decreases during task performance by experts [59]. Observations of calculations by experts have reported increased activity in the medial frontal gyrus, parahippocampal gyrus, anterior cingulate gyrus and right-middle-occipito-temporal junction, as well as the left paracentral lobule [60]. Other studies of arithmetical expertise have shown larger regions of increased activity [61]. These results suggest that experts utilize different or more extensive brain pathways. However, other studies suggest that more skilled subjects make more efficient use of brain regions and activity. In these circumstances, less brain activity is observed on cognitive tasks. On the other hand, those who struggle to perform cognitive tasks often have to recruit more brain areas as a compensatory mechanism [62]. The present study's results suggest that level of expertise may have an important effect on brain function. There was a trend towards an inverse correlation between change in complexity and CBF alteration in each region. Since these correlations were inverse, the implication is that greater increases in complexity were associated with progressively decreased CBF in each region. This interesting finding taking into account the complexity of the texts psychographed deserves future investigations and elucidative hypotheses. One might speculate that these findings were related to those for improvisational music performances, in which decreased activity in some attentional areas have been involved in a training-induced shift toward inhibition of stimulus-driven attention, enabling a more goal-directed performance state that aids in the emergence of spontaneous creative activity [63], [64]. Additionally, a recent study showed that alcohol intake, which decreases frontal lobe activity, appears to improve creativity [65]. However, improvisational music performance and alcohol consumption states are quite peculiar and distinct from psychography. Future research is needed to thoroughly compare psychography to other similar states and more precisely elucidate the relationship between frontal lobe function and depth, intensity, and complexity of written content produced in this interesting mediumistic state.

Overall, the fact that experienced mediums had lower rCBF than less expert mediums may be due to their having more years of practice and doing more psychographies per month (Table 1, Figure 1). However, considering the experts' high complexity scores for their psychographed content, it is not clear whether the decreased brain activity is related to more efficient brain function during the task, or the influence of other variables.

Although aware of problems in conceptualizing trance, for the purpose of this study we used a more consensual and phenomenological definition of trance proposed by Cardeña [66]: a temporary alteration of consciousness, identity, and/or behavior evidenced by at least two of the following: (1) marked alteration of consciousness; (2) narrowed awareness of immediate surroundings; (3) movements experienced as being beyond one's control. In qualitative terms, since there is no one single expression of mediumship but rather important differences between people and occasions, our subjects reported varying types of “spiritual contact”. The less expert mediums were emotionally affected and reported feeling inspired during psychography, and being in a semi-conscious state – phrases came to them as if dictated – in relation to the written content, whereas the experienced mediums said that they were “out of their bodies” and had no control over the content “elaborated by the spirit”. The superior temporal gyrus, which contains the auditory cortex, was activated during psycography for less expert mediums, who heard phrases as if they were being dictated, but deactivated in the experienced subjects, who had no conscious control over the psychographed content. The superior temporal gyrus is also involved in linguistic comprehension and is a key area related to auditory hallucination in psychotic patients [49].

Decreased activity of the left prefrontal cortex, which is involved in categorizing and rating experience [3], [67] may be partly related to the subjective account of dissociative trance as reported by the experienced mediums, and is consistent with the notion of automatic writing rather than planning written content. Studies of language processing consistently show involvement of the superior temporal cortex and precentral gyrus as crucial for processing words and their hypoperfusion results in selective impairment of written work [46], [47], [68]. Consistent activation in these areas during writing is expected in healthy subjects. These regions were hypoactivated in the experienced subjects' brains during psychography, and they did not show the impaired written text we would expect with hypoactivation presented [46], [47], [68]. The lower level of activity in the temporal cortex and precentral gyrus, as well as the hippocampus and anterior cingulate in experienced mediums lends support to their subjective reports of being unaware of content written during psychography. It should be noted that no changes in CBF were observed in the caudate nuclei previously described in glossolalia. Subjects also showed a reduced CBF in the right prefrontal cortex, and these discrepancies may be related to different processing language-related tasks during these trance-like states [25].

Subjects attributed their trance writing to “spirits”. Compared to normal writing, less expert mediums showed more activation in the same cognitive-processing areas during psychography, whereas experienced mediums showed a significantly lower level of activation (Figure 1). The less expert ones had to “work harder”, as shown by their relatively higher levels of activation of the cognitive processing area during psychography. Experienced mediums showed significantly reduced rCBF changes during psychography, which is consistent with the notion of automatic (non-conscious) writing and their claims that an “outer source” was planning the written content. Brain regions known to be involved in planning writing were activated less, even though the content was more elaborate than their non-trance writing (Table 3). These findings are not consistent with faking or role-playing, both of which have been offered as explanations for psychography. Planning-related neural circuits would presumably be recruited to compose more elaborate texts if the subjects were faking trance states (Figure 1, Table 4). On the contrary, studies of cognitive-processing regions involved in reasoning and planning written content [46]–[48] showed decreased activity in the experienced mediums, who reported that they were not conscious of psychographed content and had no control over it. Subjects reported that their trance state involved a “relaxed state of mind”. The state of relaxation alone might explain the lower overall activity of the brain, but the fact that subjects produced complex content in a trance dissociative state suggests they were not merely relaxed. Moreover, relaxation seems an unlikely explanation for the underactivation of brain areas specifically related to the cognitive processing being carried out. As the first step toward understanding the neural mechanisms involved in non-pathological dissociation, we emphasize that this finding deserves further investigation both in terms of replication and explanatory hypotheses.

In non-pathological conditions, a person may benefit from these dissociative abilities, although such a disposition may develop into dissociative pathology after adverse/traumatic events [3]–[5]. The absence of current Axis I or II mental disorders [14] in the groups is in line with current evidence that dissociative experiences are common in the general population and not necessarily related to mental disorders, especially in religious/spiritual groups [16]. Mediums' blood-flow alterations differed between experienced and less expert subjects, which highlights the diversity of the dissociative phenomenon in healthy subjects, and suggests that further research should address criteria for distinguishing between healthy and pathological dissociative expressions in the scope of mediumship.

A limitation of this study arises from the small sample size, which obviated the detailed analysis that a larger sample could support. We only used a threshold for clusters as a correction for significance since correction for multiple comparisons would be over-conservative for this exploratory study. However, in a larger study, we could run a more robust analysis to correct for multiple comparisons, as well as small volume correction. Neither did we perform a single-subject analysis since we considered this study to be exploratory, and therefore simplified the analysis to random-effects in an attempt to determine basic differences between groups.

Different lines of research are coming together in a promising development pointing toward more profound comprehension of consciousness and dissociation [7]–[9], [17], [31]. Although the study of spiritual experiences such as mediumship is seminal to the development of our current understanding of the mind, their relevance was neglected by researchers in the past century [10], [19]. The present study provides useful preliminary data and points to the potential utility of epistemologically informed in-depth studies of dissociative states of consciousness and spiritual experiences to improve our understanding of the mind and its relationship with the brain.
